# Zebrafish Models of Rare Neurological Diseases like Spinocerebellar Ataxias (SCAs): Advantages and Limitations

**DOI:** 10.3390/biology12101322

**Published:** 2023-10-10

**Authors:** Sreeja Sarasamma, Anwarul Karim, James P. Orengo

**Affiliations:** 1 Departments of Neurology and Neuroscience, Baylor College of Medicine, Houston, TX 77030, USA; 2 Department of Fisheries and Wildlife, Michigan State University, East Lansing, MI 48824, USA; 3 School of Clinical Medicine, Li Ka Shing Faculty of Medicine, The University of Hong Kong, Hong Kong SAR, China

**Keywords:** zebrafish, neurodegeneration, spinocerebellar ataxia, behavioral assays, drug discovery

## Abstract

**Simple Summary:**

Zebrafish can be used as an effective model for studying various genetic neurological disorders, such as spinocerebellar ataxias. They are cost effective, easy to genetically manipulate, transparent in the larvae stage allowing for in vivo imaging, and amenable to rapid compound screens. Zebrafish can link the gap between neuro-specific drug discovery and clinical trials.

**Abstract:**

Spinocerebellar ataxia (SCA) is a heterogeneous group of rare familial neurodegenerative disorders that share the key feature of cerebellar ataxia. Clinical heterogeneity, diverse gene mutations and complex neuropathology pose significant challenges for developing effective disease-modifying therapies in SCAs. Without a deep understanding of the molecular mechanisms involved for each SCA, we cannot succeed in developing targeted therapies. Animal models are our best tool to address these issues and several have been generated to study the pathological conditions of SCAs. Among them, zebrafish (*Danio rerio*) models are emerging as a powerful tool for in vivo study of SCAs, as well as rapid drug screens. In this review, we will summarize recent progress in using zebrafish to study the pathology of SCAs. We will discuss recent advancements on how zebrafish models can further clarify underlying genetic, neuroanatomical, and behavioral pathogenic mechanisms of disease. We highlight their usefulness in rapid drug discovery and large screens. Finally, we will discuss the advantages and limitations of this in vivo model to develop tailored therapeutic strategies for SCA.

## 1. Spinocerebellar Ataxias (SCAs)

Spinocerebellar ataxias (SCAs) are a specific group of central nervous system (CNS) degenerative diseases that lead to progressive ataxia or loss of balance and coordination as their main clinical manifestation. They are a genetically diverse group, consisting of about 50 genes with disease-causing mutations, and exhibit an autosomal dominant mode of inheritance [1,2]. SCAs are categorized into three major groups: those caused by dynamic repeat expansion mutations (repeat expansion SCAs), those caused by non-repeat mutations, and those with currently unknown underlying gene mutations. Clinically, SCAs manifest with core symptoms of progressive cerebellar ataxia, dysarthria, and oculomotor nystagmus. However, due to their genetic diversity, they also display a heterogeneous mix of additional clinical features, such as pyramidal and extrapyramidal signs, peripheral neuropathy, and cognitive dysfunction [2].

SCAs caused by dynamic repeat expansion mutations can be further divided into those with mutations in coding regions and those in non-coding regions. Expanded repeat SCAs, whether in the coding or non-coding region of the disease gene, are known as dynamic mutations because they exhibit repeat instability and tend to expand during germline transmission, a phenomenon referred to as genetic anticipation. Eight SCAs have repeat expansions in a coding region (SCA-1, 2, 3, 6, 7, 8, 17, and DRPLA), each of which contains CAG repeats, resulting in expanded polyglutamine tracts in their respective proteins. These toxic polyglutamine-expanded proteins have been shown to form intracellular/intranuclear aggregates within neurons, and their presence correlates with transcriptional dysregulation, oxidative stress, neuronal excitotoxicity, and apoptosis. A clear polyglutamine length threshold exists, beyond which the pathology becomes fully penetrant, and the longer the polyglutamine expansion, the earlier the onset of disease and the severity of symptoms. Within this group of CAG repeat expansion ataxias, SCA8 is unique, in that polyserine and polyalanine repeats produced from repeat-associated non-AUG translation have been detected, in addition to toxic polyglutamine protein [3,4]. Furthermore, there may be a role for toxic RNA in pathogenesis from the CTG expansion transcript generated in the 3′UTR of *ATXN8OS* (opposite strand of *ATXN8*) [5]. Six SCAs are caused by dynamic repeat expansion mutations in non-coding regions, such as introns or the 5′/3′ untranslated regions (UTRs) (SCA-10, 12, 27B, 31, 36, and 37). In these cases, the repeat nucleotide expansion varies from a tri- to hexanucleotide repeat unit. Non-coding repeat expansions are thought to disrupt the splicing of essential genes as well as RNA toxicity [1,6]. There are 29 SCAs caused by other mutations, such as missense, nonsense, frameshift, and splice site mutations. While dynamic repeat expansion SCAs are typically present in adulthood, many SCAs in this “other mutation” group manifest in childhood and can even appear as early as infancy. Five SCAs (SCA-4, 9, 18, 30, and 32) either have not had their chromosomal regions determined, or the precise genes where the mutations reside remain unknown [2].

Neuroimaging studies in SCA patients have revealed a loss of gray matter volume in multiple brain regions, not just the cerebellar hemispheres [7]. Most SCA animal studies have been conducted in mouse models, focusing on providing a deeper understanding of cerebellar Purkinje neuron pathogenesis and pathophysiology. In terms of molecular studies, the most progress has been made on SCAs with polyglutamine expansions. Here, the pathology is complex, involving both toxic gain of function of the mutant protein and loss of the normal protein function [1,8,9]. One hallmark of all polyglutamine disorders is the intracellular accumulation of mutant protein fragments. Various studies have shown the importance of the subcellular localization of the mutant protein, protein folding, the role of nuclear inclusions, and clearance and post-translational modification in disease progression [1,9,10,11]. Interestingly, the polyglutamine proteins in SCAs are generally ubiquitously expressed, which contrasts with the selective neuronal degeneration seen in their disease course. Degradation of polyglutamine-expanded proteins may occur through autophagy or the ubiquitin–proteasome system, and dysfunction of these critical protein recycling pathways might also be involved [12].

Dominant ataxias currently lack effective treatments [10], but animal models are playing a critical role in unraveling the cellular and molecular mechanisms underlying these diseases to aid the development of potential therapies. While mouse models have been extensively employed due to their similarities to humans in terms of nervous system function and related genes, they come with limitations [13] and require substantial resources and costs. Other organisms like *Caenorhabditis elegans* and *Drosophila melanogaster* have also been explored as potential models for SCAs, but their non-vertebrate nature and significant differences in nervous system structure limit their suitability. The pressing need for a more comprehensive understanding of SCA pathophysiology highlights the urgency to identify efficient animal models that can facilitate the development of effective therapeutic strategies. Zebrafish, with their unique characteristics and a nervous system that exhibits significant homology to humans, have emerged as a highly promising animal model for investigating neurological diseases. This article provides a comprehensive review of the current knowledge and future possibilities offered by zebrafish as a successful model for translational research of human neurodegenerative diseases, with a distinct focus on SCAs.

## 2. Similarities and Differences in the Zebrafish and Human Brain

Considerable progress has been made in our understanding of the larval and adult zebrafish nervous systems at various levels, including the gross, cellular, molecular, and circuit levels. While there is a striking homology in the basic organization of the CNS between humans and zebrafish, there are also key differences. The forebrain, midbrain, and hindbrain structures of the developing brain can be visually distinguished by 24 h post-fertilization (hpf) in the zebrafish embryo [14]. The forebrain develops into the telencephalon, diencephalon, hypothalamus, and the retina. The mature zebrafish telencephalon, like mammals, has two hemispheres. However, in zebrafish, the hemispheres are covered by a T-shaped ventricle, whereas in mammals, the hemispheres enclose three central ventricles [15]. Zebrafish telencephalon contains the pallium (dorsal telencephalon), the subpallium (ventral telencephalon), and the olfactory bulb. The zebrafish pallium can be further divided into the dorsal, medial, lateral, and ventral pallium. Although structurally different, these divisions correspond topologically to the mammalian isocortex, amygdala, hippocampus, and piriform cortex, respectively. On the other hand, the subpallium in zebrafish corresponds to the mammalian subcortical area that includes basal ganglia [14,15,16]. The zebrafish diencephalon contains the thalamus, pineal body, and habenula [14]. Similar to mammals, the telencephalon is involved in the regulation of social behavior, memory, and emotion. On the other hand, the diencephalon is responsible for regulating functions such as attention, alertness, and circadian behaviors [14]. The precise understanding of the neural circuits and molecular mechanisms underlying these cognitive functions and behaviors in zebrafish remains incomplete and is a subject of active investigation. The zebrafish midbrain, which is crucial for vision and hearing, contains optic tectum and tegmentum. The tectum is analogous to superior colliculus in mammals [14]. Similar to mammals, the cerebellum and medulla oblongata are present in the zebrafish hindbrain; however, the pons is absent [6].

The neurotransmitter systems found in the zebrafish brain, including GABA, glutamate, dopamine, noradrenaline, serotonin, histamine, and acetylcholine, are reminiscent of those present in the mammalian brain, highlighting the similarities between the two [17,18]. At the cellular level, the central nervous system of zebrafish contains all the glial cell types—astrocytes, oligodendrocytes, and microglia—similar to humans, alongside neurons. Although the details regarding the morphological, molecular, and functional heterogeneity of these neuronal and glial cells, as well as their interactions across different brain regions, are still poorly understood, efforts to delineate the complexity have already begun to emerge at the whole-brain atlas level [19,20]. These ongoing efforts and a better understanding of the complexity of the zebrafish brain at different stages of development would have significant implications for disease modeling and screening of candidate genes, especially in the current era of rapid advancements in the discovery of disease-related genes, including those associated with neurodevelopmental and neurodegenerative disorders.

When it comes to studying spinocerebellar ataxias, the cerebellum, particularly the Purkinje cells within it, has received significant attention and is considered one of the most extensively researched and comprehended brain regions. Much of this research has been conducted using mouse models, which have provided valuable insights into the understanding of spinocerebellar ataxias. The cerebellum displays noticeable gross anatomical differences between zebrafish and humans. In humans, the cerebellum is extensively folded, resembling a cauliflower on sagittal sections. These folds allow for the identification of separate lobes. In zebrafish, the cerebellum, which develops from rhombomere 1, has a smooth surface and is located in the upper back part of the hindbrain. Once differentiated, zebrafish cerebellum can be divided into three major compartments. The primary component is the corpus cerebelli, which is evolutionarily linked to the spinocerebellum in humans involved in motor coordination. Anteriorly, the corpus cerebelli transitions into the valvula cerebelli, which is not present in humans. Posteriorly to the corpus cerebelli is the lobus caudalis along with the laterally located eminentia granularis, collectively referred to as the caudolateral lobe. This region is homologous to the vestibulocerebellum (flocculonodular lobe) in humans, which is responsible for body posture and balance control. Despite the markedly different gross morphology of zebrafish cerebellum compared to the human cerebellum, the general cellular architecture shows great homology to that of humans [21]. The neuronal organizations in the zebrafish cerebellum can be described in three layers. The innermost layer, known as the granule cell layer, primarily consists of cerebellar interneurons and serves as the input layer. The outermost layer, called the molecular layer, is predominantly composed of neuropil and houses the majority of cerebellar synaptic connections, thus functioning as the processing layer. The intermediate layer, known as the Purkinje cell layer, serves as the output layer. Additionally, while the zebrafish cerebellum lacks the presence of organized deep cerebellar nuclei, as observed in humans, the eurydendroid cells found in the zebrafish cerebellum are considered to be functionally equivalent to the neurons of deep cerebellar nuclei [22]. While the major cell types found in the human cerebellum are conserved in the zebrafish cerebellum, certain cell types such as basket cells and unipolar brush cells have not been reported in the zebrafish cerebellum [21].

As discussed previously, zebrafish hold great potential as a model system for studying candidate genes and conducting high-throughput drug screens for neurodevelopmental and neurodegenerative diseases, including spinocerebellar ataxias. However, it is crucial to consider the similarities and differences in brain organization between humans and zebrafish moving forward. This will ensure the accurate interpretation of neuroanatomical, cellular, molecular, and behavioral changes observed in zebrafish resulting from experimental manipulations.

## 3. Zebrafish Are an Emerging Model for the Study of SCAs

The zebrafish has a fully characterized genome that displays a high degree of similarity to humans. At least 80% of genes associated with human diseases listed in the Online Mendelian Inheritance in Man (OMIM) database are conserved in zebrafish [23]. This makes zebrafish an advantageous model organism for studying gene function and its role in human diseases through various gene manipulation techniques that result in either transient or stable genetic alterations. Zebrafish serve as a valuable intermediary between in vitro research and more expensive mammalian research, such as those involving mice, balancing physiological complexity and experimental throughput. The design and connectivity of the human CNS are more closely related to zebrafish than to simpler model organisms like flies or worms.

Compared to mice, zebrafish’s larvae–adult duality, faster development, and longer lifespan make them an attractive choice for modeling the progression of neurodegenerative diseases. Their externally developing, transparent embryos facilitate easier genetic manipulation and analysis of individual neuron activity during normal and pathological conditions [24]. Furthermore, the patterning, pathfinding, and connectivity within the zebrafish CNS have been studied well and show strong parallels with humans [25], making them a powerful tool for investigating neuronal diseases. As such, numerous neurological disorders have been modeled in zebrafish, including amyotrophic lateral sclerosis, autism spectrum disorder, Alzheimer’s disease, Parkinson’s disease, Huntington’s disease, prion-related diseases, and epilepsy [26]. As zebrafish become increasingly popular in biomedical research, there is a growing interest in utilizing them to enhance our understanding of cerebellar diseases.

Zebrafish are especially relevant for studying SCAs because their cerebellar organization and function closely resemble those of mammals. Moreover, their cerebellum develops, differentiates, and matures during the transparent embryonic and larval stages [21], making zebrafish an excellent in vivo system for bioimaging. Their genetic tractability enables the combination of molecular genetic studies with in vivo imaging of cellular and physiological processes using a wide array of fluorescent proteins and biosensors [27,28,29]. SCA-related research in zebrafish can be divided into two main approaches. In one approach, the disease gene is either knocked down or knocked out using tools like morpholinos or CRISPR/Cas systems, regardless of the specific mutation present in patients (Table 1). These studies primarily aim to understand gene function in vivo and have been crucial in elucidating the roles of relevant genes, offering valuable insights into the pathophysiology of SCAs where loss of function is the primary disease driver. In the other approach, the mutated gene, such as a polyglutamine-expanded gene, is introduced into the zebrafish (Table 1). While more challenging than simple gene silencing strategies, these studies may provide a more accurate disease model and valuable insights into the pathophysiology of gain-of-function mutations driving disease progression. For instance, Elsaey et al. recently developed a zebrafish model for SCA1 [30]. SCA1 results from CAG repeat expansion, which leads to a polyglutamine tract expansion in the Ataxin-1 protein. This model was established through Purkinje cell-specific expression of a human patient-derived pathological Ataxin-1 containing 82 non-interrupted glutamine residues. In this SCA1 model, the researchers observed progressive, age-related degeneration of Purkinje cells. Additionally, the model exhibited impaired exploratory behavior in a novel tank test assay, correlating with Purkinje cell degeneration. Intriguingly, the study also revealed that the rostral regions of the zebrafish cerebellum were more susceptible to Purkinje cell degeneration than the caudal regions. In another study, Watchon et al. established a zebrafish model of SCA3 by expressing pathological human Ataxin-3 containing 84 glutamine residues [31]. This model revealed decreased survival and motor impairment in zebrafish. These studies elegantly illustrated the feasibility of using zebrafish for modeling SCAs caused by repeat expansions. However, such models are still lacking for other SCAs caused by repeat expansions.

## 4. Behavioral Assessments of Zebrafish

Zebrafish, similar to humans, demonstrate a diverse range of cognitive processes and behaviors, including anxiety, fear, learning, memory, perception, social skills, and sleep patterns [41]. However, while SCAs can impact cognitive functions in humans, the core symptoms of SCAs primarily manifest as impairments in motor functions and coordination. These symptoms often include a progressive loss of balance and coordination, oculomotor abnormalities, and slurred speech [1,2]. It is important to note that zebrafish do not exhibit many of these human behaviors and phenotypes associated with SCAs. For instance, expecting zebrafish to display slurred speech is unrealistic, as speech is not a characteristic of their biology. Furthermore, zebrafish maintain posture in an aquatic environment, which differs fundamentally from how humans maintain balance. Additionally, zebrafish do not possess limbs with sophisticated fine motor skills. Therefore, it is unrealistic to expect zebrafish to manifest the same phenotypic manifestations of SCAs observed in humans. However, this does not diminish the value of using zebrafish as a model organism to study SCAs and other neurodegenerative diseases. Indeed, a growing number of well-developed tests and paradigms are now accessible for studying zebrafish behaviors, some of which have been used in studying SCAs [42,43,44]. A simple analysis of swimming behavior alone can offer an integrated assessment of stimulus detection by sensory systems, transmission through central processing systems, and activation of the motor system [42]. Consequently, these behavioral assessments function as screening tools to determine if genetic manipulations lead to behavioral disturbances and if these changes can be mitigated or reversed through intervention with chemicals or other means.

A range of behavior parameters, mainly focused on swimming, locomotion, and exploratory behavior, can be conducted in larvae and adult zebrafish to comprehend the mechanism and progression of ataxia in SCAs. In recent times, the evolution of software solutions, including solutions from Etho Vision “www.noldus.com (accessed on 29 August 2023)”, Loligo (www.loligosystems.com (accessed on 29 August 2023)”, Loco scan (www.cleversysinc.com (accessed on 29 August 2023)”, and video tracking software like idTracker (www.idtracker.es (accessed on 29 August 2023)”, has significantly facilitated the assessment of precise and subtle movements. A multitude of behavior assays in both larvae and adult zebrafish highlight the zebrafish as an eminent model for investigating neurodegenerative disorders including SCAs. Some of these assays are summarized below.

### 4.1. Three-Dimensional (3D) Locomotion Tracking

Zebrafish larvae demonstrate proficient swimming skills at 4–5 days post-fertilization (dpf) once their swim bladder has developed. The complex locomotion observed in zebrafish is the result of coordinated interactions among different types of neurons, particularly the reticulospinal neurons located in the brainstem, working in conjunction with descending projections in the vestibulospinal and neuromodulatory pathways [45]. It is important to note, however, that although these neural pathways are conserved across various vertebrate species, zebrafish lack the corticospinal and rubrospinal tracts [46], which are crucial for motor movement in humans. Yet, locomotion tracking and swimming behavior provide useful phenotypic information on the neuromuscular functions resulting from perturbations in any component within the complexly integrated neuromuscular circuits. For instance, in a recently developed zebrafish SCA1 model containing a patient-derived pathological mutant Ataxin-1 with 82 non-interrupted glutamine residues, there is a reduced exploratory behavior attributed to advanced Purkinje cell loss [30].

To record the swimming behavior of zebrafish, the animals are placed on an optical stage equipped with an infrared light source. A mirror, tilted at a 45-degree angle, is positioned at the top border of the tank to create a 3D image reflection. Prior to video recording, the zebrafish are given 5 min to acclimate to the new environment. The zebrafish behaviors can then be recorded for an additional 5 min. Subsequently, individual trajectories are extracted from the recorded video images using idTracker [47]. Representative swimming behavioral parameters that can be assessed include total moving distance, average angular velocity, average speed, and meandering movement. Assays commonly used to characterize locomotion and neuromuscular deficits in zebrafish include the swim tunnel assay, touch-evoked escape response, and birefringence.

### 4.2. Swim Tunnel Assay

The swim tunnel assay is an important tool for studying locomotor behavior in zebrafish. This assay involves inducing forced swimming by placing zebrafish within a swim tunnel, a device that generates a controlled laminar water flow [48]. By leveraging the principle of rheotaxis, which is the inherent behavioral response of aquatic organisms to swim against the water flow to maintain their position [49], the swim tunnel assay enables the analysis of various parameters. These parameters include critical swimming speed, which represents the maximum velocity a fish can sustain for a specific duration, and burst/coast swimming, a behavior that conserves energy by alternating between short bursts of swimming against the current and gliding downstream [48]. The assay allows for the assessment of locomotor behavior under the influence of genetic abnormalities, altered environmental conditions, or specific compounds. While commercially available systems (e.g., from Loligo systems), despite being relatively expensive, can enhance reproducibility across laboratories through standardization, there are also detailed protocols available for constructing swim tunnel systems in-house at a significantly lower cost [48]. These in-house systems offer an alternative means to ensure reproducibility across different research facilities.

### 4.3. Touch-Evoked Escape Response

Spontaneous muscle contractions in zebrafish embryos become noticeable at approximately 17 h following fertilization, and their responsiveness to tactile stimulation emerges at around 24–27 hpf [50,51]. Studying alterations in these movements offers valuable insights into potential neuromuscular impairments. The touch-evoked response assay focuses on measuring the maximum acceleration during a burst swimming motion triggered by an external stimulus. To conduct this assay, zebrafish embryos (e.g., at 2 dpf) are dechorionated and placed individually in Petri dishes filled with embryo medium with a high-speed camera positioned above the dish. Video recording is initiated, and a gentle touch is applied to the top of the embryo’s head using a blunt needle, which serves as a mechanosensory stimulus. The fish’s response is captured and the recorded videos are subsequently analyzed using software that tracks the fish’s trajectory and quantifies the maximum acceleration during the burst swimming motion [52]. If conducted at 6 dpf, tactile stimuli can be induced by touching the tail region of the larvae [53].

### 4.4. Birefringence

The birefringence assay [54] is utilized to assess the structural integrity of skeletal muscle in zebrafish. Skeletal muscle is innervated by motor neurons which are affected in some SCAs. This assay, which is non-invasive and straightforward, capitalizes on the phenomenon of light rotation as it traverses ordered matter, such as the pseudo-crystalline arrangement of muscle sarcomeres. To conduct this assay, tricaine is used to anesthetize zebrafish embryos (3–7 dpf) for easier positioning. The embryos are then placed between two polarized light filters on a dissecting microscope, and the resulting birefringence phenotype is observed. To quantify birefringence, images of both wild-type and mutant fish are captured under polarized light using the same exposure settings and magnification. Subsequently, software like ImageJ can be used to analyze these images. The significance of this assay lies in its capacity to offer insights into the condition of myofibrillar organization in live zebrafish embryos. Wild-type zebrafish, possessing well-organized skeletal muscle, exhibit a pronounced brightness against the dark background. Conversely, muscle mutants display distinctive birefringence patterns that correspond to the particular muscular disorder they represent.

### 4.5. Exploratory Behavior

A novel tank test can be used to assess stress response and exploratory behavior in zebrafish [55]. When exposed to an unfamiliar environment, zebrafish demonstrate decreased exploratory movement and reduced speed, which serve as indicators of anxiety levels. To conduct this test, the fish are initially acclimated to the test room and then placed in the novel tank for a specific duration while their swimming activity is recorded [56]. Subsequently, the recorded videos can be examined to determine various parameters, including the frequency of zebrafish entering the top section of the novel tank, the duration of time spent in the top or bottom areas, the distance covered in the top or bottom regions, the time taken to enter the top section, the swimming velocity, the total distance traveled, and details regarding movement patterns and instances of freezing [56]. It is crucial to provide comprehensive information about the specific conditions under which the experiments are conducted in order to enhance reproducibility as simple variations such as the direction of light source can lead to potential variations in the obtained results [55].

### 4.6. Light–Dark Test

The light–dark preference test is based on the natural tendency of an animal to explore or avoid specific areas within a given environment [57,58]. This test has been applied in zebrafish neuroscience research for investigating anxiety-like behavior, relying on the innate preference of adult zebrafish for a black compartment over a white one [59]. Although variations in the test protocol exist, generally, the assay involves a brief acclimation phase followed by unrestricted exploration of a tank containing both black and white compartments, which can be recorded using a camera [59,60]. Different variables, including the duration spent in the white compartment, the time taken to enter the white compartment, the number of entries into the white compartment, the duration of these entries, erratic swimming, freezing episodes, and thigmotaxis can be analyzed. Adult zebrafish typically exhibit a preference for the dark compartment, and treatments inducing anxiogenic effects tend to intensify this preference. Additionally, it can also increase thigmotaxis, erratic swimming, and freezing in the white compartment [59]. The test can also be adapted for use with larval fish, which could prove advantageous for conducting high-throughput phenotypic assessments and drug screening [57]. Another related approach involves evaluating zebrafish behavior through the alternation of light and dark conditions. To implement this method, zebrafish larvae are placed in a multi-well plate and given a 20 min period to acclimate. Subsequently, the larvae are exposed to light for a few minutes, followed by a few minutes of darkness. The total distance covered by the larvae during each phase can be measured and subjected to analysis [61].

### 4.7. Optokinetic Response

The optokinetic response is a visual reflex observed in zebrafish larvae, which develops as their visual system matures. It can be reliably measured by the fourth day after fertilization [62]. This response is characterized by slow eye movements that track the visual stimulus, followed by rapid resetting movements when the eyes reach their movement limit. By adjusting the stimulus parameters such as speed, contrast, and spatial frequency, it is possible to evaluate visual acuity and sensitivity [42]. To conduct the assay, zebrafish larvae are first immobilized on a Petri dish. The optokinetic response can then be triggered by presenting a stimulating movie through a movie projector, displaying vertical black and white stripes that move horizontally at a consistent speed, projected onto a circular screen [56]. Alternatively, the optokinetic response can be induced by placing the larvae in the center of a motor-driven drum with vertical stripes on its inner surface, allowing for rotation and creating a moving visual stimulus for the larvae [62]. A camera is used to record the eye movement responses to the stimulus, which can then be analyzed using various parameters, including the extent and velocity of the eye movement and the number of saccades. Other protocols for measuring the optomotor response in juvenile and adult zebrafish are also available [63,64], which can be valuable for studying diseases that might affect the visual system after development, such as certain SCAs [65,66].

## 5. Imaging Studies in Zebrafish

Significant advancements have been made in brain imaging technologies, including 3D imaging techniques, which can be effectively utilized in modeling and studying spinocerebellar ataxias (SCAs) and other neurodegenerative diseases in zebrafish. While the human brain is incredibly complex, containing billions of neurons, the adult zebrafish brain, with approximately 10 million neurons [67], and the larval zebrafish brain, with between 80,000 and 100,000 neurons [68], provide a simpler model for imaging the nervous system and investigating neurological disorders, as their general brain organization and functions are largely conserved. The combination of behavioral assays with imaging holds immense potential for examining neurodegenerative diseases. Researchers have utilized stabilized or partially stabilized larvae in conjunction with multiphoton and light-sheet fluorescence microscopy (LSFM) to capture images of genetically encoded calcium indicators (GECIs) in various behaviors, including opto-motor control, auditory and visual responses, olfaction, and prey-capture decisions [67]. Additionally, techniques that can monitor whole-brain neural activity in freely swimming larval zebrafish have been described recently [69]. A recent study utilizing zebrafish transgenic line marking astrocytes demonstrated the potential of zebrafish as a highly promising and innovative model system for studying the development and functioning of astrocytes in vivo [70], highlighting the exceptional value of zebrafish in advancing our understanding of astrocyte biology that can be leveraged in studying the neurodegenerative diseases where astrocytes are being increasingly recognized as a crucial player.

There has been a growing emphasis among researchers to investigate various brain functions at the level of neural circuits, with the goal of identifying “engrams”—groups of neurons that encode specific experiences through their activity patterns and synaptic connections. Various imaging techniques, such as selective plane illumination microscopy, structured illumination microscopy, and light field microscopy, enable whole-brain activity mapping in zebrafish larvae [68]. Larval zebrafish offer an alternative to rodents in such systems’ neuroscience studies because it allows for comparatively easier delineation of neural circuity [71,72], providing valuable insights into the engrams involved in both normal and abnormal behavioral manifestations. While such experiments typically involve head-tethered fish, the development of tracking microscopes holds significant potential for conducting similar investigations in freely swimming fish [69], as well as to expand the research in investigating much broader and complex behaviors.

Zebrafish are well suited for high-throughput imaging experiments due to the abundance of embryos available for experimentation and the ease of imaging compared to rodents. The Vertebrate Automated Screening Technology (VAST BioImager) enables high-throughput screening in zebrafish [73]. Additionally, the development of 3D digital brain atlases for zebrafish [19], essential tools in modern neuroscience research, allows visualization of the brain’s three-dimensional structure and facilitates the study of genetic perturbations or drug effects. Furthermore, an interactive atlas of the circuit architecture of the larval zebrafish brain has been created, providing cellular-resolution insights [74]. Therefore, with these state-of-the-art imaging technologies and resources, zebrafish have emerged as an attractive model system for widespread adoption in studying SCAs and neurodegenerative conditions in the near future.

## 6. Zebrafish for the Development of Therapeutic Approaches

The search for therapeutic approaches to treat SCAs has been challenging. As a result, there is presently no FDA-approved treatment available, even though clinical trials are currently in progress [1,2,10,75]. There is a pressing need to develop effective disease-modifying treatments for SCAs that can alleviate symptoms and modify the course of the disease. Recent advancements in understanding the pathophysiological mechanisms of SCAs have suggested promising avenues for both symptomatic relief and disease modification. Common pathophysiological themes in SCAs include proteotoxicity, RNA toxicity, ion channel dysfunction, impaired bioenergetics, loss of nuclear integrity, and compromised DNA repair mechanisms [1,10,11]. To develop and test disease-modifying therapies for SCAs, it is crucial to gain a detailed understanding of these mechanisms, discover new disease-associated pathways, and importantly, model these pathophysiological processes in animals. Arguably, the most compelling therapeutic targets for SCAs are approaches that target the upstream or root causes of these pathological cascades [10]. In this regard, correcting the mutation or reducing the expression of the mutated gene presents an attractive strategy for treating these diseases. The advancements in genome editing technologies, particularly CRISPR/Cas system, hold great promise for developing therapies that can correct mutations or silence the expression of mutated genes. Various approaches such as antisense oligonucleotides, small interfering RNAs, short hairpin RNAs, and artificial microRNAs can be utilized to reduce the expression of the mutated gene or a key regulator of the mutated gene’s expression [10]. Additionally, virus-based gene delivery can be considered for SCAs where the disease pathogenesis is contributed to by the loss of function resulting from a mutation. While mouse models have been and will continue to be instrumental in disease modeling, as well as the development and preclinical testing of novel therapeutic approaches, zebrafish also offer significant potential due to advances in applying these techniques, their suitability as high-throughput model systems, and the increasing availability of zebrafish neuroscience research tools and resources.

In addition to gene silencing approaches, another potential strategy is to identify compounds that can reduce the levels or toxicity of mutated gene products or rescue the disruption in downstream biological processes that underlie specific SCAs. Zebrafish are valuable for conducting large-scale screenings of small molecules, aiding in the detection of substances capable of inducing or ameliorating neurodegenerative phenotypes in the brain and spinal cord. The availability of various neurobehavioral phenotyping assays and the transparency during early development allow for easy study of the effects of drug treatments on disease progression. Transgenic zebrafish lines with promoter-driven expression of fluorescent proteins or whole-mount immunodetection methods enable the correlation of molecular changes with behavioral impairments. For instance, a recent study [61] explored the therapeutic potential of a novel calpain inhibitor, BLD-2736, for treating SCA3 using a transgenic zebrafish model. The study demonstrated that administering BLD-2736 to SCA3 zebrafish larvae between 1 and 6 dpf resulted in improved swimming performance and reduced the presence of insoluble protein aggregates. Another important aspect of zebrafish is that its BBB undergoes maturation between 3 and 10 dpf and shares structural and functional similarities with the mammalian BBB [76]. Therefore, zebrafish offer a rapid and reliable in vivo system for predicting the BBB permeability of novel compounds.

## 7. Conclusions

**Figure 1:**
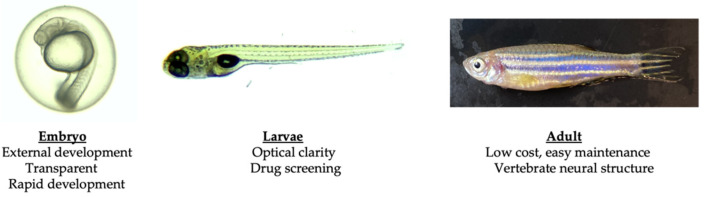
Advantages of using zebrafish as a SCA model organism.

In summary, the zebrafish’s close genetic, neuroanatomical, and neurochemical resemblance to humans, combined with their rapid development, ease of genetic manipulation, optical transparency during early development, and high-throughput behavioral and drug screening capabilities, make them an outstanding model for exploring the fundamental mechanisms of SCAs and developing potential therapeutic strategies (Figure 1). The availability of diverse behavioral assays, advanced imaging techniques, and a growing body of tools and resources in zebrafish neuroscience further amplifies the potential of zebrafish in advancing our understanding of these rare disorders.

However, it is important to recognize the differences between zebrafish and the human brain as well. Additionally, researchers need to be aware that the phenotypic measurements captured by zebrafish might not represent the complexity observed in humans. Being aware of the differences and limitations will eventually be helpful in understanding which aspects of the diseases can be reliably modeled and investigated in zebrafish. For instance, while zebrafish offer a promising model for studying SCAs due to the similarities between their cerebellum and that of humans, it is important to note that certain subtle movement abnormalities, such as impairment in motor coordination and imbalances observed in many SCAs, may not be fully recapitulated in zebrafish models. Therefore, the widely used locomotor activity readouts, which have been primarily used for evaluating motor deficits, might not truly represent the phenotypic abnormalities observed in humans. Similarly, cognitive function assays in zebrafish may not fully capture the complexity of cognitive impairments seen in humans. However, the behavioral abnormalities observed in these assays while studying SCAs may still reflect similar underlying pathogenic and neurobiological mechanisms in humans and thus might be useful. Once phenotypic abnormalities are detected, they could be further investigated at the molecular, cellular, or circuit level in the brain to dissect the basis of the abnormal behavioral manifestations. Moreover, it is recognized that SCAs involve structures beyond the cerebellum and can impair non-cerebellar functions, such as motor neuron degeneration observed in SCA1 patients and SCA1 mouse models [77,78]. Zebrafish can be a useful tool for investigating and identifying therapeutic options for such abnormalities, and these studies can benefit from knowledge obtained from other studies and methodologies investigating these structures in other disorders, such as motor neuron disease modeling in zebrafish.

## Figures and Tables

**Table 1:** Studies on SCAs genotypes and phenotypes using Zebrafish animal model.

Type of SCAs	Human Gene	Mutation Generated	Method	Phenotype Behavior	References
**SCA1**	*ATXN1*	CAG (82) repeat expansion	Stable integration of humans *ATXN1* with CAG repeats driven by bidirectional Purkinje cell-specific expression system	Exploratory behavior impaired and Purkinje cell degeneration during young adult stage	[30]
**SCA2**	*ATXN2*	CAG (30) repeats in ATXN2 together with knock down of *C9orf72*	Transient ubiquitous expression of human *ATXN2* with CAG repeats together with antisense morpholino knockdown of zebrafish *C9orf72* endogenous gene	Reduction in locomotor activity, disrupted arborization, and shortening of the motor neuron axons	[32]
**SCA3**	*ATXN3*	CAG (84) repeat expansion	Double transgenic fish carrying Huc:Gal4 and UAS: human *ATXN3*-CAG (84) fused to GFP	Motor dysfunction in adult zebrafish and reduced axonal length	[31]
**SCA6**	*CACNA1A*	-	Morpholino mediated knockdown of *cacna1ab*	Loss of touch evoked swimming	[33]
**SCA7**	*ATXN7*	-	Morpholino mediated knockdown of *atxn7*	Impaired Purkinje and granule cell differentiation	[34]
**SCA13**	*KCNC3*	Missense human R420H, R423H or F363L, zebrafish R335H	Bidirectional Purkinje cell specific expression system; F0 transgenesis driven by Purkinje or motor neuron promoters; or endogenesis gene knockin	Eye movement deficits, Neuronal degeneration in early larval stages, alterations in Purkinje cell firing rate, and abnormal motor neuron axon morphology and firing rate.	[35,36,37,38]
**SCA37**	*DAB1*	ATTTC (58) repeat expansion	mRNA microinjection	Impairs early embryonic development	[39]
**SCA49**	*SAMD9L*	Missense S626L	mRNA microinjection	Impaired mobility and sensory functions	[40]

## Data Availability

Not applicable.
